# A review of China’s compensation program for adverse reactions following immunization

**DOI:** 10.3389/fpubh.2023.1211976

**Published:** 2023-08-07

**Authors:** Wu Yingxiong

**Affiliations:** School of Health Economics and Management, Nanjing University of Chinese Medicine, Nanjing, China

**Keywords:** adverse reaction following immunization, compensation system, Corona virus disease 2019, vaccine administration law, vaccine injury

## Abstract

Vaccination is a compelling measure to battle infectious diseases and protect public health. However, because of the constraints on human cognition, it is difficult to ensure that vaccines are safe. Adverse reactions to immunization can cause individual injury. In numerous countries, no-fault programs have been established to compensate individuals for vaccine-related injuries. China also established a vaccine injury compensation system with its own unique characteristics. The Vaccine Administration Law was promulgated in 2019 to establish a compensation system for those who experience adverse reactions following immunization; nevertheless, the compensation system is imperfect. Even when the applicable terms are applied to deal with vaccine-related injuries, some issues remain, such as unreasonable diagnosis and evaluation procedures for adverse reactions, excessively strict standards regarding proof and inconsistent compensation standards across the country. Therefore, to provide effective compensation for vaccine recipients, it is important to clarify the standards of proof and establish a sensible vaccine injury compensation system that includes Corona Virus Disease 2019 vaccine-injury compensation.

## 1. Introduction

The 2019 novel coronavirus (SARS-CoV-2) is a new coronavirus affecting humans that emerged at the end of December 2019 in Wuhan, China. Globally, as of 29 March 2023, there have been 761,402,282 confirmed cases of Corona Virus Disease 2019 (COVID-19), including 6,887,000 deaths (1). Effective vaccines are crucial for ending the global pandemic. More than 90 vaccines against SARS-COV-2 are being evaluated in clinical trials, and several have been approved for large-scale vaccination (2). However, for all of the novel coronavirus vaccines, the time from the non-clinical to the clinical stage and then to the market has been short. These vaccines are all emergency measures taken by humans in response to the novel coronavirus. According to statistics released by the Chinese Centre for Disease Control and Prevention (China CDC), as of 30 April 2021, there have been 31,434 adverse events following immunization (AEFI) reported to China CDC, adverse reactions accounted for 17.04% of adverse events, and the ratio of serious adverse reactions was 0.07 per 100,000 doses, indicating that severe adverse reactions are extremely rare at less than 1 per 10,000 doses (3).

Some issues had been also observed with other vaccines. A study shows that the proportion of 226,320 AEFI cases (including 43 vaccines) in males was higher than that in females, and the highest proportion of AEFI occurred in 0–1 years, as shown in Table 1 (4). The top ten vaccines with the incidence of reported adverse reactions were MR，LepV, MMR, HZV, COVID-19 V, PPCV13¸DTaP-Hib/IPV¸DTaP-Hib, MPCV-AC, and MPV-ACYW, as shown in Table 2 (4). The adverse reactions were mainly anaphylactic (e.g., allergic shock), neurological (e.g., febrile convulsion) and BCG-specific reactions, the incidence of anaphylactic reactions (2.15/100,000) including allergic shock, allergic rash, angioedema, laryngeal edema, Arthurs reaction, and others was the highest, of which 77.60 and 17.62%, respectively, occurred within 1 day and 1–3 days after vaccination (4). In addition, serious adverse reactions following immunization were rare.

**Table 1:** Gender and age distribution of reported AEFI cases in China in 2020.

Variable	Total AEFI	
	Number of cases	Constituent ratio (%)
Gender	-	-
male	120,736	53.35
female	105,584	46.65
Age	-	-
0–1	153,726	67.92
2–6	52,239	23.08
7–17	4,721	2.09
≥18	15,634	6.91

**Table 2:** Top 10 vaccines with the incidence of reported adverse reactions in China in 2020 (per 100,000 doses).

Vaccine	Adverse reaction	
	Number of cases	Incidence
MR	1,150	18.49
LepV	1	17.07
MMR	3,550	14.16
HZV	14	11.69
COVID-19 V	516	8.82
PPCV13	382	6.38
DTaP-Hib/IPV	201	4.11
DTaP-Hib	95	2.78
MPCV-AC	152	2.55
MPV-ACYW	112	2.25

Nevertheless, serious adverse reactions following immunization can cause considerable physical and mental damage to individuals. In general, vaccination benefits public health, and those who experience injury due to vaccination should not be responsible for dealing with the consequences on their own. If adversely affected vaccine recipients are not reasonably compensated, it may intensify conflicts between doctors and patients and even influence social safety and stability in China (5). Therefore, it is imperative not only to strengthen research and technological innovation to minimize the probability of adverse reactions, especially serious adverse reactions, but also to establish an effective compensation system. Because an effective vaccine-injury compensation system can improve immunization rates and reduce diseases to benefit public and community health. The Vaccine Administration Law established a vaccine injury compensation system to ensure that individuals who experience adverse reactions (6) to vaccinations are compensated. However, there are some problems with the current vaccine injury compensation system. This article addresses these questions by reviewing and analyzing the legal framework of China’s vaccine injury compensation system to enable vaccine recipients to obtain compensation more swiftly, efficiently, and fairly.

## 2. Methods and framework

### 2.1. Methods

To describe how China’s government compensates individuals injured by vaccines and problems with the vaccine injury compensation system, the study examines the literature covering national surveillance data on AEFI, the vaccine management system, and tort law. To analyze the status and issues of the vaccine injury compensation system, a narrative review was performed (7). A narrative approach was preferred to a systematic one since the main concern was not to address a narrowly focused question and then summarize data (7). The main aim was that of improving the current vaccine injury compensation system to enable vaccine victims to obtain compensation more swiftly, efficiently, and fairly. This necessitated clarifications and insights into the status and issues of the vaccine injury compensation system, and there was a need for a more interpretative and descriptive synthesis of existing literature, thereby requiring a narrative review (7).

The literature search was undertaken on PubMed, CNKI, and the following institutional websites: World Health Organization (WHO) (link: https://www.who.int); China CDC (link: https://www.chinacdc.cn/jkzt/ymyjz/); National Center for ADR Monitoring, China (link: https://www.cdr-adr.org.cn/); China Judgments Online (link: https://wenshu.court.gov.cn/website/wenshu/181029CR4M5A62CH/index.html). The literature was limited to documents published in Chinese and English. The types of documents accepted were journal articles, legal documents, books, and websites of relevant organizations. The knowledge domains included: (1) vaccine injury surveillance data, (2) China’s vaccine injury compensation system (e.g., diagnosis procedures, standards of proof, and compensation standards), and (3) vaccine injury compensation systems around the globe.

### 2.2. Framework

In this study, we provide an overview of vaccine injury compensation systems around the globe and review the compensation system for adverse reactions following immunization in China. Then, based on describing the limitations and problems of China’s vaccine injury compensation system, we focus on provisions for the improvement of the vaccine injury compensation system, which include lowering the standards of proof for legal causation and establishing a reasonable vaccine injury compensation system.

## 3. Results and discussion

### 3.1. Overview of global practices

Historically, the primary mechanism for vaccine-injury compensation has been a tort system whereby victims must file a tort lawsuit against the manufacturer. However, litigation is adversarial, protracted, and uncertain (8). Continued reliance on the tort system may be unjust. In response, some national governments have established vaccine injury compensation programs (VICPs) as an alternative solution to this problem. Such VICPs were instituted based on the ethical principle of compensatory justice, which states that governments have a special obligation to compensate individuals injured by a vaccination that is for the benefit of the public (9). At present, 25 member states of the World Health Organization (WHO), including Austria, France, Germany, Canada, Italy, Japan, and the United States, have established VICPs to compensate individuals who experienced serious vaccine-related injuries (10). The VICPs of 15 of these member states are administered at the central government level, while others are implemented at the provincial level (e.g., Italy and Germany) or by the insurance sector (e.g., Sweden). Compensated funds are generally provided by the government and pharmaceutical companies (11). All compensation schemes provide a lump sum of money covering medical costs, disability benefits, and death benefits (12); moreover, they also all require proof demonstrating a causal link between vaccination and injury (11), as shown in Table 3 (13). In general, most compensation schemes operate on a standard of proof that is more liberal than the legal standard, which is also known as the ‘balance of probabilities’ approach, and requires a burden of proof that is less strict than ‘beyond a reasonable doubt’ (14). For example, in the United States, to simplify determinations of causality, a vaccine-injury table was created to determine in advance if a certain vaccine injury is eligible for compensation (15).

**Table 3:** Comparison of compensation patterns for adverse reactions following immunization.

Item	Finance pattern	Fund pattern	Insurance pattern	Mixed pattern
Management entity	Government	Government	Non-governmental organization	Government
Capital source	Government budget	Vaccine tax	Premium paid by the manufacturers	Government finance and manufacturers
Covered vaccine types	Routine or mandated vaccines	Taxed vaccines	vaccines produced by the insured manufacturers	Routine or mandated vaccines
Compensation content	Injury with reasonable evidence related to vaccination/limited to rare and severe disability or death	Injury (including disability and death) with reasonable evidence related to vaccination	Injury (including disability and death) and mental damage with reasonable evidence related to vaccination	Injury with reasonable evidence related to vaccination/limited to rare and severe disability or death
Typical country/region	France, Denmark, Italy, Germany, United Kingdom	United States, Taiwan	Sweden	Japan, Finland, Norway

Although the existing no-fault VICPs in 25 countries do not directly cover injuries related to COVID-19 vaccines (16), some countries have activated their emergency compensation programs. In the United States, a VICP was created to compensate individuals who develop medical issues associated with certain vaccines (17). However, the declaration of a public health emergency by the Department of Health and Human Services in March 2020 required that individuals who suffer adverse reactions from a COVID-19 vaccine must file compensation claims through the countermeasures injury compensation program (CICP) (18). Moreover, the CICP is far less generous and less accessible than the VICP. It compensates individuals for only the most serious injuries, requires a higher burden of proof than the VICP, and limits awards for damages (19). The WHO has also established a new no-fault compensation program for rare yet serious adverse events associated with COVID-19 vaccines. This program was initially financed through Gavi COVAX AMC donor funding, and it provides fair, no-fault, lump sum compensation to eligible individuals in 92 low- and middle-income countries and economies who suffer certain serious adverse events after receiving a COVID-19 vaccine distributed through the COVAX Facility until 30 June 2022 (20). This is the only global vaccine injury compensation system and significantly reduces the need for recourse to the law courts, a potentially lengthy and costly process (21).

### 3.2. Review of China’s compensation system for adverse reactions following immunization

In the 1980s, China began to compensate individuals injured by vaccines; however, it had not yet established a comprehensive compensation system. In 1980, the Ministry of Health (MOH) rolled out interim rules for adverse reactions and accidents following immunization, stipulating that victims’ medical costs would be covered by reimbursement provided by one’s medical and labor insurance as well as payments by the local government. In 1982, the MOH promulgated the Regulations on the Work of National Planned Immunization, stating that the local government would pay the medical costs for victims. These two laws did not stipulate specific compensation methods, procedures, quotas, or standards, and they only provided compensation for medical costs. In practice, compensation decisions have mainly been decided by the local government according to local governments’ health spending budgets (22). However, due to the lack of local government funding for health expenditures for a considerable period, victims were unable to obtain reasonable compensation when they had an adverse reaction to a vaccine (22).

In 2005, the State Council of the People’s Republic of China (hereafter, ‘State Council’) issued the Regulation on the Administration of Circulation and Immunization of Vaccines (hereafter, ‘Vaccines Circulation Regulation’), which established a no-fault compensation program for class 1 vaccines (compulsory vaccinations) and class 2 vaccines (non-compulsory vaccination). It also stipulated the remedy methods and the source of compensation. The Vaccines Circulation Regulation also requires provincial governments to establish specific compensation measures. As of 29 October 2015, China’s 32 provincial governments, including those of the five autonomous regions, four municipalities directly under the central government, and Xinjiang Production and Construction Crops, have established local compensation regulations and policies (23). Most provincial compensation schemes provide for basic expenses, disability benefits, and death benefits. In addition, the State Council introduced a commercial insurance compensation mechanism for those who experience adverse reactions following immunization in the revision of the Vaccines Circulation Regulation (12). Governments and vaccine manufacturers can voluntarily purchase commercial insurance to compensate vaccine-injury victims.

In 2019, the Vaccine Administration Law reclassified class 1 and class 2 vaccinations into immunization program vaccines (compulsory vaccinations) and non-immunization program vaccines (non-compulsory vaccination), respectively. The law also encourages compensating vaccine recipients who experience adverse reactions following immunization using commercial insurance and other means. Moreover, according to the law, compensation shall be given when one experiences an adverse reaction following immunization and when the possibility cannot be ruled out. This is the first adverse reaction compensation system to be established by Chinese law (6).

### 3.3. Problems with the vaccine injury compensation system in China

#### 3.3.1. Unreasonable diagnosis and evaluation procedures of adverse reactions

To make a compensation claim for vaccine injury in China, one must obtain an official diagnosis and have an evaluation of one’s adverse reaction following immunization. Although these procedures seem simple, it is difficult for vaccine recipients to receive compensation. First, the evaluation of adverse reactions is arranged by provincial and municipal medical associations to protect the rights of all parties; however, receiving an official diagnosis is a prerequisite for having one’s adverse reactions evaluated, and this can increase the time it takes to evaluate adverse reactions. Ultimately, it takes too much time for those making claims to be compensated (24). The local China CDC office oversees the diagnosis of adverse reactions and supplies vaccines to the vaccinating agency. In this way, the China CDC is both the referee and the athlete. Diagnoses are often not accepted by vaccine recipients. In such cases, the vaccine recipient’s only option is to appeal to the municipal (provincial) medical association where the vaccinating agency is located for a revaluation (25). Second, the experts who serve on panels overseeing evaluations and diagnoses are active in related disciplines, such as clinical medicine and epidemiology in the area, and they easily overlap. Therefore, evaluations can be affected by the diagnoses. As a result, unreasonable procedures and the composition of expert panels may infringe on vaccine recipients’ rights.

#### 3.3.2. Excessively strict standards of proof

For vaccine recipients, proving the legal causation between vaccination and injury is crucial for obtaining compensation. In China, the compensation scheme applies a strict standard of proof that is generally based on epidemiological causation (12). The expert panel excludes other various factors before determining that a patient has experienced an adverse vaccination reaction. That is, in the appraisal of adverse reactions, experts exclude each of the six situations stated by the Vaccine Administration Law and then make their final evaluation. Nevertheless, tort law distinguishes between two stages of causation in medical negligence: factual and legal causation. In China, the main test for factual causation is the ‘but-for’ test (26), and the judgment of legal causation adopts the ‘adequate causation’ theory (27). Moreover, ‘adequacy’ refers to whether a behavior is sufficient to produce the claimed harm, and in this process, the reasonable foreseeability of the victim’s damage plays an important role.

However, the courts do not apply lenient legal standards when determining causation and directly accept experts’ diagnoses and evaluations. Only 34.18% of vaccine disputes regarding adverse events following immunization litigated in Chinese courts resulted in compensation being awarded (28). Victims are often told that the injury and vaccination are coincidental. Even if primary people’s courts adopt a generous standard of proof, the intermediate people’s courts will still overturn their judgments based on official diagnoses and evaluations. In the case of *Cao Mingzhi and Yang Xueqin v. Wuhan Biological Products Research Institute Co., Ltd. and Aimei Hissen Vaccine (Dalian) Co., Ltd.*, the autopsy and pathological examination results showed that the diagnosis was consistent with thymic lymphoid dysplasia, and it did not exclude the possibility that the injection of the vaccine contributed to the progression of the disease. However, according to the clinical manifestations, autopsy and pathological examination results, vaccine quality inspection results, and more, it was concluded that although vaccination could not be excluded as one of many inducing factors, there was no direct causal link between the vaccination and death; therefore, it was judged to be a coincidence. The primary people’s court believed that the diagnosis established ‘no direct causation’, and not ‘no causation’. Given the diagnosis as well as the fact that the vaccination was one of the strongest external stimuli known before Cao’s sudden death, it was highly probable that the vaccination-induced Cao’s death (29). Therefore, the court adopted preponderant probability standards to determine causation, and it did not directly accept the diagnosis.

However, upon appeal, the intermediate people’s court overturned the judgment of the primary people’s court regarding the causation of the adverse reaction, writing, ‘Although Cao died after being vaccinated, the cause of his death was the thymic lymphoid constitution, which has no direct causation with immunization. It is not an adverse reaction and is a coincidence. Based on the above facts, there is no legal causation between Cao’s death and vaccination. The primary people’s court improperly determined the causal association involved in the case, and this court corrects it (30). The two courts’ contrary determinations of causation stem from their different methods of examining the evaluation. The primary people’s court analyzed the evaluation based on the physical condition and time between vaccine administration and death. Meanwhile, the intermediate people’s Court did not consider other factors when examining the evaluation. An analysis of the rationale of the two courts reveals that the viewpoint of the primary people’s court is preferable. The evaluation demonstrated that the deceased would not have died if he had not been vaccinated and factual causation was established. The next stage is to determine whether legal causation was established. The facts identified by the primary people’s court showed that the vaccination was one of the strongest external stimuli known before Cao’s sudden death, which meets the requirement of ‘adequacy’. Moreover, there is reasonable foreseeability that vaccinations can harm vaccine recipients with the thymic lymphoid constitution; therefore, legal causation was established.

#### 3.3.3. Inconsistent compensation standards across the country

In China, there are disparities in the amount and scope of compensation as well as compensation methods (23). Except for the provinces of Tianjin, Guangdong, and Fujian, which provide fixed death payments, all provinces and autonomous regions calculate death compensation based on the *per capita* disposable income of an urban resident of the province in the previous year (31). Because the *per capita* disposable income of an urban resident in each province is different, there is a large gap in the amount of compensation regarded for vaccine recipients’ injuries, even though they are caused by the same or similar adverse reaction following immunization. For example, in Jiangsu and Anhui provinces in 2021, the maximum death compensation was equal to the *per capita* disposable income of an urban resident multiplied by 20 times. In 2021, the *per capita* disposable income of an urban resident in Jiangsu was 57,743 Yuan (32), and the maximum death compensation in Jiangsu was 1,154,860 Yuan. In 2021, the *per capita* disposable income of urban residents in Anhui was 43,009 Yuan (33), and the maximum death compensation was 860,180 Yuan. Therefore, in 2021, the difference in death compensation between the two neighbouring provinces was 294,680 Yuan. This describes the typical situation of ‘same life but different prices’ among residents of different provinces (34). Although the State Council determines the compensation scope, standards, and procedures for adverse reactions following immunization, and although the provinces formulate specific implementation measures, the problem of ‘same life but different prices’ is difficult to completely solve due to the differences in the level of economic development among provinces.

#### 3.3.4. Difficult claims and low insurance confidence

Commercial insurance can benefit social risk-sharing mechanisms. However, regarding the compensation for adverse reactions following non-immunization program vaccines, provinces requiring vaccine companies (or MAHs) to purchase vaccination-accident insurance for non-immunization program vaccines may place additional burdens on companies and ultimately increase vaccine prices (35). In addition, there are other problems with vaccine-related commercial insurance in China, such as complicated procedures and other barriers to making insurance claims (36). Specifically, vaccine recipients still have to go through the application, evaluation, verification, and insurance claim processes to obtain insurance compensation (37), and denial during any of these procedures can result in a vaccine recipient being uncompensated. These problems have diminished confidence in vaccine-related commercial insurance in China. According to a survey, the total coverage rate of commercial supplementary insurance for children with adverse reactions is 53%. A reason for this is that some parents do not trust commercial insurance (38). Thereby, an effective vaccine injury compensation system is necessary for the overall wellbeing of children (39).

## 4. Conclusion and recommendation

### 4.1. Using a generous standard of proof for causation

The Vaccine Administration Law includes circumstances that cannot be excluded from compensation for adverse vaccination reactions. It also stipulates that the scope of compensation is subject to the compensation catalog and shall be adjusted based on actual situations. In 2020, the National Health Commission issued the 2020 Reference Catalog and Description of the Compensation Scope for Adverse Vaccination Reactions (hereafter, ‘Reference Catalog’), which represents a positive development in the determination of the causes of adverse vaccination reactions. However, the Reference Catalog is only a reference; therefore, even if the vaccine recipient meets the conditions listed in the Reference Catalog, this does not mean that causation has been established between vaccination and injury.

This differs from the National Childhood Vaccine Injury Act in the United States. This act created a VICP and funding for the program is provided through an excise tax placed on covered vaccines (40). The legislation that established the VICP also created a vaccine-injury table that lists the illnesses, disabilities, injuries, and conditions that are covered, as well as the time it takes for a vaccine recipient to experience their first symptom or the onset of significant aggravation after vaccine administration (41, 42). That is, the injuries listed on the vaccine-injury table must occur within a pre-determined period after vaccination for the vaccine to be covered by the VICP, and the nature of the injuries must comply with the vaccine-injury table or the definitions and descriptions in the auxiliary explanation section. Moreover, if the injury conforms to the above conditions, it is presumed that it was caused by the vaccine (43, 44). The direct presumption that any injury on the list has been caused by the vaccine lowers the standard of proof and shortens the time it takes to receive compensation. Therefore, to expand the scope of compensation and enhance the immunization coverage rate in China, causation between vaccination and personal injury should be legally recognized as long as the case is in the Reference Catalog. Moreover, the personal injury should be presumed to be caused by an adverse reaction to simplify procedures for the diagnosis and evaluation of adverse reactions. This, in turn, will enable victims to receive compensation promptly.

In addition, if an injury caused by vaccination is not in the Reference Catalog, it is difficult for vaccine recipients to prove causation between the undetectable potential risk of the vaccine and the personal injury. Therefore, when determining whether an adverse reaction has been caused by immunization, the preponderant probability standard should be adopted. Since vaccination incidents are a type of medical incident, the theory of preponderant probability can also apply to the determination of causation (45). Therefore, as long as there is a correlation between a vaccine injury and vaccination, the government or MAHs should compensate vaccine recipients for adverse reactions to mitigate against negative publicity (46) unless it can be determined that the injury was not caused by vaccination.

### 4.2. Improving the compensation system of the immunization program vaccine injury

In China, injecting immunization program vaccines is not only a citizen’s right but also a duty. Such programs are led by the government and benefit the public welfare. Therefore, the government is justified in mandating vaccines to increase the vaccination rate and protect public health (12). Under these circumstances, an administrative and legal relationship has been formed between vaccine recipients and the government. The government, as the main body that provides compensation for those harmed by immunization programs (13), must assume compensation liability and implement a unified national compensation standard to solve the problem of ‘same life but different prices’ caused by the vaccination program.

In addition, it should be noted that the government also assumes the responsibility of compensating vaccine recipients who suffer COVID-19 vaccine injuries (8), especially if the government restricts specific individual rights for the collective good (47). Specifically, the COVID-19 vaccines were promoted by governments at all levels under the direction of the central government (48). In addition, many people, especially those with professional obligations, such as healthcare workers, are required to get vaccinated (49), and the government also recommends other high-risk citizens receive COVID-19 vaccines, such as the older adult and school-age children. Individuals who are required or have been urged to receive the COVID-19 vaccination, primarily for the benefit of others and society, should have access to fair and timely compensation (50).

More specifically, for cases of adverse reactions and cases that cannot be ruled out as adverse reactions, the Chinese Government can learn from Taiwan’s experience to establish a differentiated compensation standard, as the two jurisdictions have similar legal systems and cultures. In Taiwan, determinations of causality between vaccination and injury are assessed by the Vaccine Injury Compensation Working Group, and they are classified into three types: unassociated, associated, and indeterminate. The amount of compensation for an ‘associated’ and ‘indeterminate’ relationship between vaccination and injury is different. For death compensation, if the evaluation determines that the relationship is ‘associated’, then the amount of compensation is NT$500,000– NT$6,000,000. If the evaluation finds the relationship to be ‘indeterminate’, then the range of compensation is NT$300,000– NT$3,500,000 (51). This compensation standard is clear and simple to implement. Compensation methods for vaccine injuries in mainland China should specify that the compensation amount for adverse reactions following immunization must be higher than that for adverse reactions where the cause is indeterminate.

### 4.3. Establishing compensation funding for non-immunization program vaccine injury

Non-immunization program vaccines are vaccines received voluntarily by residents, mainly to meet individual needs. These differ from immunization program vaccines, which are overseen by the government and provided free of charge. Immunization program vaccinations can be regarded as a government initiative, while non-immunization program vaccinations are personal choices. The government should not be responsible for providing vaccine-injury compensation. Moreover, owing to the defect of commercial insurance implementation, it also does not apply to non-immunization program vaccine-injury compensation in China. Therefore, a vaccine injury compensation fund should be established (52).

The government can draw on the experience of Taiwan to establish a vaccine injury compensation fund. In Taiwan, vaccine manufacturers and importers must pay a fee, which funds Taiwan’s Vaccine Injury Compensation Program (53). A levy of NT$1.5 should be collected for each dose of vaccine. The central competent authority can adjust the levy when the balance of the fund falls below NT$150 million or exceeds NT$400 million. Compared to the sources of commercial insurance, the sources of vaccine injury compensation funds are relatively stable and most suitable for establishing a centralized vaccine injury compensation system. Vaccine companies or MAHs should pay the vaccine injury compensation fund according to an assessed standard, and a unified compensation standard should be implemented to ensure that victims receive equal compensation for the same or similar adverse reactions following immunization.

## 5. Limitation

With the increase in the rate of vaccination, the issues of adverse reactions to immunization and obtaining effective compensation have attracted increasing public attention (54). In this context, the significance of this study is to survey and improve China’s vaccine injury compensation system for those suffering from adverse reactions to enhance public confidence and benefit public health. Nevertheless, some limitations should be noted. First, the national surveillance information on AEFI was up to 2021, which did not affect the research results, because the study mainly interpreted and analyzed the vaccine injury compensation system. Second, the study was focused on the improvement of China’s vaccine injury compensation system, and the mechanisms of the adverse events resulting from different vaccinations were not of great concern. Third, the study adopted a narrative review rather than a systematic review, thereby providing interpretation and critique rather than summarizing data.

## Author’s note

1. Adverse drug reactions are those in which a causal relationship has been established, while adverse drug events are those in which a causal relationship has not been established.
2. In 2013, Ministry of Health changed its name to National Health and Family Planning Commission of the PRC, subsequently changing its name to National Health Commission of the PRC in 2018.
3. The ‘same life but different price’ referred to the different compensation for death between urban and rural residents in China. In the article, we adopt it to explain the disparity in compensation for death among different provinces.
4. Reference Catalog and Description of the Compensation Scope for Adverse Vaccination Reactions (2020 Edition) was issued by the General Office of the National Health Commission on 7 December 2020. The Reference Catalog lists adverse reactions for 11 types of vaccines and the period for the illness onset after vaccine administration.
5. In China, the immunization program vaccination includes the national and provincial government immunization program vaccination, cluster vaccination, and emergency vaccination. The cluster vaccination refers to the organized and centralized implementation of vaccination activities in a specific range and period for specific individuals who may be infected by a certain infectious disease, and emergency vaccination refers to the preventive vaccination activities carried out for individuals who are susceptible to control the spread of the epidemic when the epidemic of an infectious disease begins or has an epidemic trend.
6. In China, immunization program vaccine injury compensation consists of commercial basic insurance and commercial supplementary insurance. Commercial basic insurance is dominated and purchased by provincial governments, and commercial supplementary insurance is voluntarily purchased by vaccine recipients.

## Author contributions

The author confirms being the sole contributor of this work and has approved it for publication.

## Conflict of interest

The author declares that the research was conducted in the absence of any commercial or financial relationships that could be construed as a potential conflict of interest.

## Publisher’s note

All claims expressed in this article are solely those of the authors and do not necessarily represent those of their affiliated organizations, or those of the publisher, the editors and the reviewers. Any product that may be evaluated in this article, or claim that may be made by its manufacturer, is not guaranteed or endorsed by the publisher.

## References

[1] World Health Organization. WHO coronavirus (COVID-19) dashboard. (2023). Available at: https://covid19.who.int/ (Accessed April 5, 2023).

[2] Fan YJ Chan KH Hung IF. Safety and efficacy of COVID-19 vaccines: a systematic review and Meta-analysis of different vaccines at phase 3. Vaccine. (2021) 9:989. doi: 10.3390/vaccines9090989, PMID: 34579226 PMC8473448

[3] China CDC. Overview of surveillance information on adverse reactions of COVID-19 vaccines in China (as of 30 April 2021). (2021). Available at: https://www.chinacdc.cn/jkzt/ymyjz/ymyjjz_6758/202105/t20210528_230911.html (Accessed April 10, 2023).

[4] Zhang L Li KL Li Y Fan CX Li Y Ren MR. Surveillance of adverse events following immunization in China, 2020. Chin J Vaccines Immunization. (2022) 28:208–18. doi: 10.19914/j.CJVI.2022041

[5] Sun YH. The discussion about the proof burden of causal relationship between mandatory vaccination and damage. Hebei Law Sci. (2015) 33:72–9. doi: 10.16494/j.cnki.1002-3933.2015.10.006

[6] Feng J Li Q. How to ensure vaccine safety: An evaluation of China’s vaccine regulation system. Vaccine. (2021) 39:5285–94. doi: 10.1016/j.vaccine.2021.07.081, PMID: 34373122 PMC8344949

[7] Greenhalgh T Thorne S Malterud K. Time to challenge the spurious hierarchy of systematic over narrative reviews? Eur J Clin Investig. (2018) 48:e12931. doi: 10.1111/eci.12931, PMID: 29578574 PMC6001568

[8] Mello MM. Rationalizing vaccine injury compensation. Bioethics. (2008) 22:32–42. doi: 10.1111/j.1467-8519.2007.00590.x, PMID: 18154587

[9] Henry LM Larkin ME Pike ER. Just compensation: a no-fault proposal for research-related injuries. J Law Biosci. (2015) 2:645–68. doi: 10.1093/jlb/lsv034, PMID: 27774216 PMC5034397

[10] D’Errico S Zanon M Concato M. “First do no harm”. No-fault compensation program for COVID-19 vaccines as feasibility and wisdom of a policy instrument to mitigate vaccine hesitancy. Vaccine. (2021) 9:1116. doi: 10.3390/vaccines9101116, PMID: 34696224 PMC8540114

[11] Mungwira RG Guillard C Saldaña A Okabe N Petousis-Harris H Agbenu E. Global landscape analysis of no-fault compensation programmes for vaccine injuries: a review and survey of implementing countries. PLoS One. (2020) 15:e0233334. doi: 10.1371/journal.pone.0233334, PMID: 32437376 PMC7241762

[12] Fei LF Peng Z. No-fault compensation for adverse events following immunization: a review of Chinese law and practice. Med Law Rev. (2017) 25:99–114. doi: 10.1093/medlaw/fwx001, PMID: 28177508

[13] Yue DH Chang J Hou ZY Wu Q Meng QY. References on international vaccination injury compensation programs. Chin Health Econ. (2014) 33:93–6. doi: 10.7664/CHE20140131

[14] Looker C Kelly H. No-fault compensation following adverse events attributed to vaccination: a review of international programmes. Bull World Health Organ. (2011) 89:371–8. doi: 10.2471/BLT.10.081901, PMID: 21556305 PMC3089384

[15] Kelly HA Looker C Isaacs D. A no-fault compensation scheme for serious adverse events attributed to vaccination. Med J Aust. (2011) 195:4–5. doi: 10.5694/j.1326-5377.2011.tb03176.x, PMID: 21728930

[16] Halabi S Heinrich A Omer SB. No-fault compensation for vaccine injury — the other side of equitable access to Covid-19 vaccines. N Engl J Med. (2020) 383:e125. doi: 10.1056/NEJMp2030600, PMID: 33113309

[17] Lateef TM Johann-Liang R Kaulas H Hasan R Williams K Caserta V. Seizures, encephalopathy, and vaccines: experience in the National Vaccine Injury Compensation Program. J Pediatr. (2015) 166:576–81. doi: 10.1016/j.jpeds.2014.10.054, PMID: 25477158

[18] Meyers PH. The trump administration’s flawed decision on coronavirus vaccine injury compensation: recommendations for changes. J Law Biosci. (2020) 7:1–11. doi: 10.1093/jlb/lsaa082, PMID: 33505704 PMC7717285

[19] Van Tassel K Shachar C Hoffman S. Covid-19 vaccine injuries — preventing inequities in compensation. N Engl J Med. (2021) 384:e34. doi: 10.1056/NEJMp2034438, PMID: 33471973

[20] WHO. COVAX no-fault compensation program. (2022). Available at: https://www.who.int/initiatives/act-accelerator/covax/no-fault-compensation (Accessed July 3, 2023).

[21] WHO. No-fault compensation programme for COVID-19 vaccines is a world first. (2021). Available at: https://www.who.int/news/item/22-02-2021-no-fault-compensation-programme-for-covid-19-vaccines-is-a-world-first (Accessed July 14, 2023).

[22] Liu P Peng XD. The adverse events following immunization (AEFI) compensation system reform in risk society. Chin J Drug Eval. (2014) 31:113–6.

[23] Wu Q Peng XD Hu XJ. Comparative analysis of provincial compensation policy of adverse reaction following immunization in China. Chin J Vaccines Immunization. (2015) 21:573–9. doi: 10.19914/j.cjvi.2015.05.021

[24] Jiao YL. Compensation for adverse reaction in preventive vaccination. Hebei Law Science. (2011) 29:31–8. doi: 10.16494/j.cnki.1002-3933.2011.05.010

[25] Cai RJ. Reform and improvement of the state vaccine injury compensation system in China. Stud Law Bus. (2021) 38:59–72. doi: 10.16390/j.cnki.issn1672-0393.2021.04.006

[26] Mcivor C. Debunking some judicial myths about epidemiology and its relevance to UK tort law. Med Law Rev. (2013) 21:553–87. doi: 10.1093/medlaw/fwt017, PMID: 23645537

[27] Huang DQ. New theory of medical law. Beijing: Law Press china (2013). 485 p.

[28] Zhang CX Tan MC Wen JX. A case study of 79 vaccine disputes of adverse event following immunization——— from the perspective of judicial decision documents. China Health Law. (2020) 28:27–34. doi: 10.19752/j.cnki.1004-6607.2020.01.006

[29] Cao Mingzhi and Yang Xueqin v Wuhan Biological Products Research Institute Co., Ltd. And Aimei Hissen vaccine (Dalian) co., ltd. (2019) Su0213 Minchu 10583. (2019) Su02 Minzhong 2245. (2019). Available at: https://wenshu.court.gov.cn/website/wenshu/181107ANFZ0BXSK4/index.html?docId=Xa7eLfpmr67yVHPeBIpW+darw6EU+qxm4/aaXABDlnG4dbsCoO3OgJO3qNaLMqsJIXT5bMV35er2P9NQnMHAjke9GTh98gpuM0smPnGSjWSe/6AydYq/FEPrNIyquswy (Accessed August 26, 2023)

[30] Cao Mingzhi and Yang Xueqin v Wuhan Biological Products Research Institute Co., Ltd. And Aimei Hissen vaccine (Dalian) co., ltd. (2020) Su02 Minzhong 2245. (2020). Available at: https://wenshu.court.gov.cn/website/wenshu/181107ANFZ0BXSK4/index.html?docId=LPj+cEzjlIM0WjyjVhBawyo8Q+hnMBQjuUbKwE6fI9CrPXZ9rDLYbZO3qNaLMqsJIXT5bMV35er2P9NQnMHAjke9GTh98gpuM0smPnGSjWSe/6AydYq/FEPrNIyquswy (Accessed August 26, 2023).

[31] Huang PH. Study on insurance compensation mechanism of rare vaccine reactions. Beijing: Capital University of Economics and Business (2019). 9–10.

[32] Jiangsu Bureau of Statistics. Statistical Bulletin of National Economic and Social Development of Jiangsu Province (2020). Available at: http://www.js.gov.cn/art/2022/3/31/art_64797_10398993.html (Accessed August 25, 2022).

[33] Anhui Bureau of Statistics. Statistical Bulletin of National Economic and Social Development of Anhui Province (2020). Available at: http://tjj.ah.gov.cn/ssah/qwfbjd/tjgb/sjtjgb/146518001.html (Accessed August 25, 2022).

[34] Zhang XD. A proper way in legal theory to crack the hard nut of similar lives but with different values. Modern Law Sci. (2008) 160:97–104.

[35] Yang Y. Expedite the enactment of the vaccine administration law and improve the compensation mechanism for adverse reactions of immunization. China Food Drug Administration. (2019) 182:4–9.

[36] Liu HH. Path selection and system conception of vaccine injury relief in China. Law Rev. (2015) 189:136–43. doi: 10.13415/j.cnki.fxpl.2015.01.012

[37] Wang Z. Research on compensation system for abnormal reaction of vaccination. Shaanxi: Northwest University (2020). 17.

[38] Xu L Zhu BQ Qian YH Zhang HM Liu Y Liang YQ. Purchase of supplementary vaccination insurance among children in Nanjing city. Chin J Vaccine Immunization. (2020) 26:336–8. doi: 10.19914/j.cjvi.2020.03.025

[39] Solarino B Nicolì S Benevento M Zedda M Oliva A. Children's health and safety: what we learned from the COVID-19 pandemic and future policy’s perspective. Front Public Health. (2023) 11:1220977. doi: 10.3389/fpubh.2023.1220977, PMID: 37346109 PMC10280161

[40] Cook KM Evans G. The National Vaccine Injury Compensation Program abstract. Pediatrics. (2011) 127:s74–7. doi: 10.1542/peds.2010-1722K 21502255

[41] Thompson KM Orenstein WA Hinman AR. Performance of the United States vaccine injury compensation program (VICP): 1988-2019. Vaccine. (2020) 38:2136–43. doi: 10.1016/j.vaccine.2020.01.042, PMID: 31982259 PMC7853082

[42] Crum T Mooney K Tiwari BR. Current situation of vaccine injury compensation program and a future perspective in light of COVID-19 and emerging viral diseases. F1000Res. (2021) 10:652. doi: 10.12688/f1000research.51160.2 35035888 PMC8733825

[43] Wang YF Yang Y. Study on vaccine injury compensation program in the United States and its enlightenments for China. China Pharm. (2020) 31:1158–65. doi: 10.6039/j.issn.1001-0408.2020.10.02

[44] Günther C Tonti L Domenici I. Vaccination as an Equaliser? Evaluating COVID-19 vaccine prioritisation and compensation. Med Law Rev. (2022) 30:584–609. doi: 10.1093/medlaw/fwac020, PMID: 36482837 PMC9732649

[45] Du YF. Causation in vaccination events in Japan. ECUPL J. (2014) 1:57–71.

[46] Attwell K Drislane S Leask J. Mandatory vaccination and no fault vaccine injury compensation schemes: An identification of country-level policies. Vaccine. (2019) 37:2843–8. doi: 10.1016/j.vaccine.2019.03.065, PMID: 31000414

[47] Meissner HC Nair N Plotkin SA. The National Vaccine Injury Compensation Program: striking a balance between individual rights and community benefit. JAMA. (2019) 321:343–4. doi: 10.1001/jama.2018.20421 30608524

[48] The State Council Information Office of the People’s Republic of China. Moderate prosperity in all respects: Another milestone achieved in China’s human rights. (2021). Available at: https://language.chinadaily.com.cn/a/202108/12/WS6114ca40a310efa1bd66868d.html (Accessed March 26 2022).

[49] Bardosh K de Figueiredo A Gur-Arie R Jamrozik E Doidge J Lemmens T. The unintended consequences of COVID-19 vaccine policy: why mandates, passports and restrictions may cause more harm than good. BMJ Glob Health. (2022) 7:e008684. doi: 10.1136/bmjgh-2022-008684, PMID: 35618306 PMC9136690

[50] Mazur A Benitez S Chuffart-Finsterwald S La Rotta R Hampton LM. COVAX no fault compensation program for COVID-19 vaccine injuries in 92 low and middle income countries. Vaccine. (2021) 39:7128–30. doi: 10.1016/j.vaccine.2021.10.047, PMID: 34732277 PMC8552561

[51] Wang PC. Updates on vaccine injury compensation program in Taiwan and program evaluation. Taiwan Epidemiol Bull. (2015) 31:149–58. doi: 10.6525/TEB.20150922.31(18).001

[52] Wang YF Yang LN Yang Y. Study on vaccine injury compensation Trust Fund in the United States and its Enlightenment to China. China Pharm. (2020) 31:1419–24. doi: 10.6039/j.issn.1001-0408.2020.12.03

[53] Chen YL Huang ASE Cheng AH Chien TJ. A comparison of vaccine injury compensation scheme in Germany, Finland and Taiwan. Epidemiol Bull. (2015) 31:159–64. doi: 10.6525/TEB.20150922.31(18).002

[54] Mungwira RG Maure CG Zuber PLF. Economic and immunisation safety surveillance characteristics of countries implementing no-fault compensation programmes for vaccine injuries. Vaccine. (2019) 37:4370–5. doi: 10.1016/j.vaccine.2019.06.018, PMID: 31213377

